# Evaluation of the tumor movement and the reproducibility of two different immobilization setups for image-guided stereotactic body radiotherapy of liver tumors

**DOI:** 10.1186/s13014-018-0962-9

**Published:** 2018-01-30

**Authors:** Constantin Dreher, Markus Oechsner, Michael Mayinger, Stefanie Beierl, Marciana-Nona Duma, Stephanie E. Combs, Daniel Habermehl

**Affiliations:** 1 Department of Radiation Oncology, University Hospital Rechts der Isar, Technical University München, Ismaningerstr. 22, 81675 Munich, Germany; 2 Institute of Innovative Radiotherapy, Helmholtzzentrum München, Munich, Germany

**Keywords:** Liver tumor, Stereotactic body radiotherapy, Patient immobilization, Robustness

## Abstract

**Background:**

The purpose of this study is to evaluate the tumor movement and accuracy of patient immobilization in stereotactic body radiotherapy of liver tumors with low pressure foil or abdominal compression.

**Methods:**

Fifty-four liver tumors treated with stereotactic body radiotherapy were included in this study. Forty patients were immobilized by a vacuum couch with low pressure foil, 14 patients by abdominal compression. We evaluated the ratio of gross tumor volume/internal target volume, the tumor movement in 4D-computed tomography scans and daily online adjustments after cone beam computed tomography scans.

**Results:**

The ratio of gross tumor volume/internal target volume was smaller with low pressure foil. The tumor movement in 4D-computed tomography scans was smaller with abdominal compression, the cranial movement even significantly different (*p* = 0.02). The mean online adjustments and their mean absolute values in the vertical, lateral and longitudinal axis were smaller with abdominal compression. The online adjustments were significantly different (*p* < 0.013), their absolute values in case of the longitudinal axis (*p* = 0.043). There was no significant difference of the adjustments’ 3D vectors.

**Conclusions:**

In comparison to low pressure foil, abdominal compression leads to a reduction of the tumor movement. Online adjustments decreased significantly, thus leading to higher accuracy in patient positioning.

## Background

Stereotactic body radiotherapy (SBRT) is able both to deliver a high radiation dose to the tumor, and to preserve sufficient liver function by little dose exposure to the surrounding liver tissue. SBRT is a therapeutic option for patients with primary and secondary liver tumors [1–5].

The liver in general is believed to have a relatively low radiation tolerance and this is a major limitation for the use of SBRT or conventionally fractionated external beam radiotherapy [6, 7]. On top of that, most patients with primary liver cancer have hepatitis B, C or alcoholic cirrhosis, resulting in an impaired liver function and even lower radiation tolerance already prior to radiotherapy. Radiation to the liver in general may result in radiotherapy-induced liver disease (RILD), possibly leading to liver failure and death and especially in case of reirradiation [8–11]. In order to guard against these events, high accuracy in patient repositioning and reduction of tumor movement due to breathing are necessary. This is of utmost importance especially in case of SBRT with high dose exposure to the liver. Due to intracorporal tumor and liver movement and difficult patient immobilization, SBRT of liver tumors is still a challenge. Intrafractional and interfractional robustness have to be improved as far as possible. Therefore, advantages and disadvantages of the following ways of patient immobilization setups have to be reevaluated: By the use of breath hold, radiotherapy mainly relies on the patients’ individual ability to control their way of breathing and can be regarded as a special way of gating. Gating in general ensures dose application only during the planned breathing cycles. There is only a small number of institutions, which can also offer tracking the target volume e.g. by the use of fiducials. Nevertheless, the implementation of fiducials is attended by a risk of complications, which is why in our institution abdominal compression is a well-established way of intracorporal immobilization. Our institution used low pressure foil until 2014 and changed patient immobilization in radiotherapy of liver tumors to abdominal compression afterwards. Both setups are used in clinical routine, but the true impact on robustness and treatment planning has not yet been evaluated. So, the present study aims to elucidate the impact of patient immobilization with abdominal compression, with regard to reducing tumor movement and increasing the accuracy in patient positioning.

## Methods

### Patient characteristics

Fifty-four patients with liver tumors, treated with SBRT at our institution between 2010 and 2016 were included in this analysis. Twenty-three and Thirty-one patients had primary and secondary liver cancer. Only patients with one lesion being irradiated were included. Fiducial markers were not implanted.

### Stereotactic body radiotherapy

**Fig. 1:**
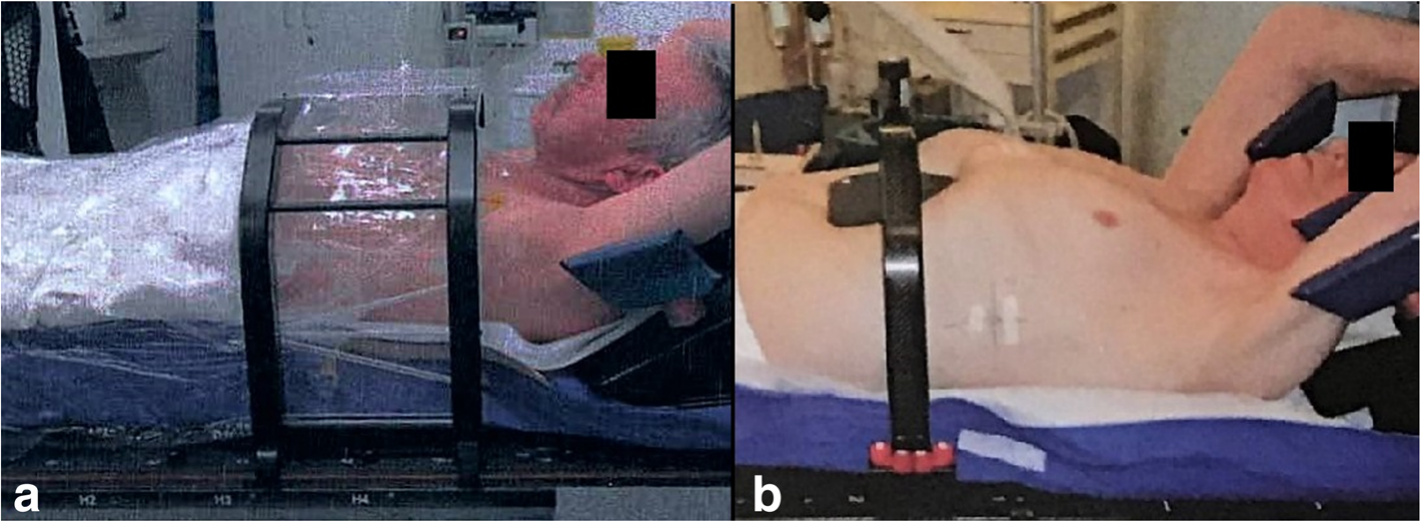
Immobilization setups. **a** Immobilization with a vacuum couch with low pressure foil. **b** Immobilization with abdominal compression

Forty patients were immobilized using a vacuum couch with low pressure foil (Medical Intelligence Medizintechnik GmbH, Schwabmünchen, Germany), 14 patients by the use of abdominal compression (Fa. ITV, Innsbruck, Österreich) (Fig. 1). The abdominal compression was performed by a rectangle plate with a triangle peak, being reproducibly placed directly under the xiphoid bone.

Computed tomography (CT) scans for treatment planning were performed with no breath-hold by a Somatom Emotion computed tomography scanner (Siemens medical solutions, Erlangen, Germany). The thickness of one treatment planning CT slice was 3 mm. Furthermore, a 4D-CT scan was carried out with the same CT scanner to evaluate the maximum extension of the tumor movement by covering the whole breathing cycle (measured by respiratory position management system (RPM, Varian Medical Systems, Palo Alto, CA)). One CT slice of the 4D-CT had the thickness of 2.1 mm. Treatment planning itself was carried out by use of the treatment planning system (TPS) Eclipse™ (Varian Medical Systems, Palo Alto, CA).

The gross tumor volume (GTV) was defined by the visible tumor on an arterial phase contrast enhanced treatment planning CT scan for primary liver cancer and on a venous phase contrast enhanced treatment planning CT scan for secondary liver cancer. The internal target volume (ITV) was defined by the maximum extension of the GTV movement in every direction in the 4D-CT scan. The planning target volume (PTV) was defined by addition of 5 mm margin in all directions.

Twenty-six patients were treated by Volumetric modulated arc therapy (VMAT) with 1 to 7 arcs. Twenty-eight patients were treated by 3D conformal radiotherapy with 6 to 11 coplanar beams. The patients’ dose prescription is summarized in Table 1.

**Table 1:** Dose prescription for 54 patients with liver tumors treated with Stereotactic Body Radiotherapy

Dose concept	Number of patients	Dose prescription	Number of patients
5 × 5Gy = 25Gy	7	• 60% Isodose • 80% Isodose	• 6 • 1
5 × 6Gy = 30Gy	3	• 60% Isodose	• 3
5 × 7Gy = 35Gy	19	• 60% Isodose • Median	• 18 • 1
3 × 7Gy = 21Gy	1	• 60% Isodose	• 1
2 × 7Gy = 14Gy	1	• 60% Isodose	• 1
5 × 7.5Gy = 37.5Gy	1	• 60% Isodose	• 1
5 × 8Gy = 40Gy	3	• 60% Isodose	• 3
3 × 12.5Gy = 37.5Gy	9	• 60% Isodose • 100% Isodose	• 8 • 1
3 × 13.5Gy = 40.5Gy	1	• 60% Isodose	• 1
3 × 15Gy = 45Gy	2	• 60% Isodose	• 2
3 × 20Gy = 60Gy	3	• 80% Isodose	• 3
14 × 3Gy = 42Gy	2	• 60% Isodose • 100% Isodose	• 1 • 1
10 × 4Gy = 40Gy	1	• 60% Isodose	• 1
10 × 5Gy = 50Gy	1	• 80% Isodose	• 1

Two patients receiving two to three fractions were scheduled to receive five fractions (5 × 7Gy = 35Gy), but the treatment was interrupted due to clinical complications. Generally, at least 700ccm of the liver should receive a radiation dose below 15Gy and the mean dose to the liver was kept as low as possible.

Treatment was performed on a Varian Clinac Trilogy linear accelerator (Varian Medical Systems, Palo Alto, CA).

Prior to each fraction image guidance was performed by a free breath cone beam computed tomography (CBCT) control scan of the liver in the immobilization setup as described before. On the basis of the manual and automatic registration of the treatment planning CT and CBCT scan the online adjustments are conducted respectively. Adjustments according to daily CBCT control scans were based on the total liver tissue together with the vertebral bones.

### Data analysis

The treatment planning volumes GTV and ITV were retrieved from the TPS Eclipse™ (Varian Medical Systems, Palo Alto, CA) and were compared for both patient groups, in order to analyze the immobilization impact on the general intracorporal movement of the liver. The mean ratio GTV/ITV was calculated to indicate the relative movement of the GTV in the ITV. Aiming at specifying the impact of the immobilization, the tumor movement in the 4D-CT scan has been analyzed. The movement has been defined as the distance between ITV and GTV on the height of the center of the GTV in the 6 directions “right lateral, left lateral, anterior, posterior, cranial, and caudal”.

Adjustments according to CBCT scan prior to each fraction of radiotherapy were analyzed, in order to evaluate the accuracy and reproducibility of immobilization: online adjustments, the absolute values of online adjustments and the summarizing 3D vector of online adjustments were analyzed. Vector calculation for each online adjustment was done by the following Eq. 1.

Equation 1. Vector equation with $\overrightarrow{\mathrm{v}}$ representing the vector and x, y and z representing its online adjustments (in the vertical, lateral and longitudinal axis), according to the cone beam computed tomography scan [cm].

$$ \overrightarrow{\mathrm{v}}=\sqrt{{\mathrm{x}}^2+{\mathrm{y}}^2+{\mathrm{z}}^2} $$

The non-parametric Mann-Whitney test was used for differences between two independent groups. Significance was defined by a level of the *p*-value below 0.05. The software SPSS (Statistical package for the Social Sciences) version 23 (SPSS Inc., Chicago, USA) was used for statistical analysis.

## Results

The GTV in the group with low pressure foil and with abdominal compression has a mean volume of 73.9ccm and 37.6ccm. The ITV in both groups has a mean volume of 94.1ccm and 67.9ccm. The mean ratio of GTV/ITV is 0.54 and 0.61 in case of low pressure foil and abdominal compression. There was no statistically significant difference between both groups (*p* > 0.05). The mean tumor movements at the height of the center of the GTV during 4D-CT are listed in Table 2. Only the cranial movement showed a significant difference between both groups (*p* = 0.02).

**Table 2:** Mean tumor movement [cm] at the height of the center of the gross tumor volume during 4D-computed tomography scan

Axis of tumor movement	Low pressure foil	Abdominal compression
Right lateral	0.54	0.48
Left lateral	0.46	0.36
Anterior	0.46	0.32
Posterior	0.48	0.44
Cranial	0.63	0.37
Caudal	0.61	0.39

**Fig. 2:**
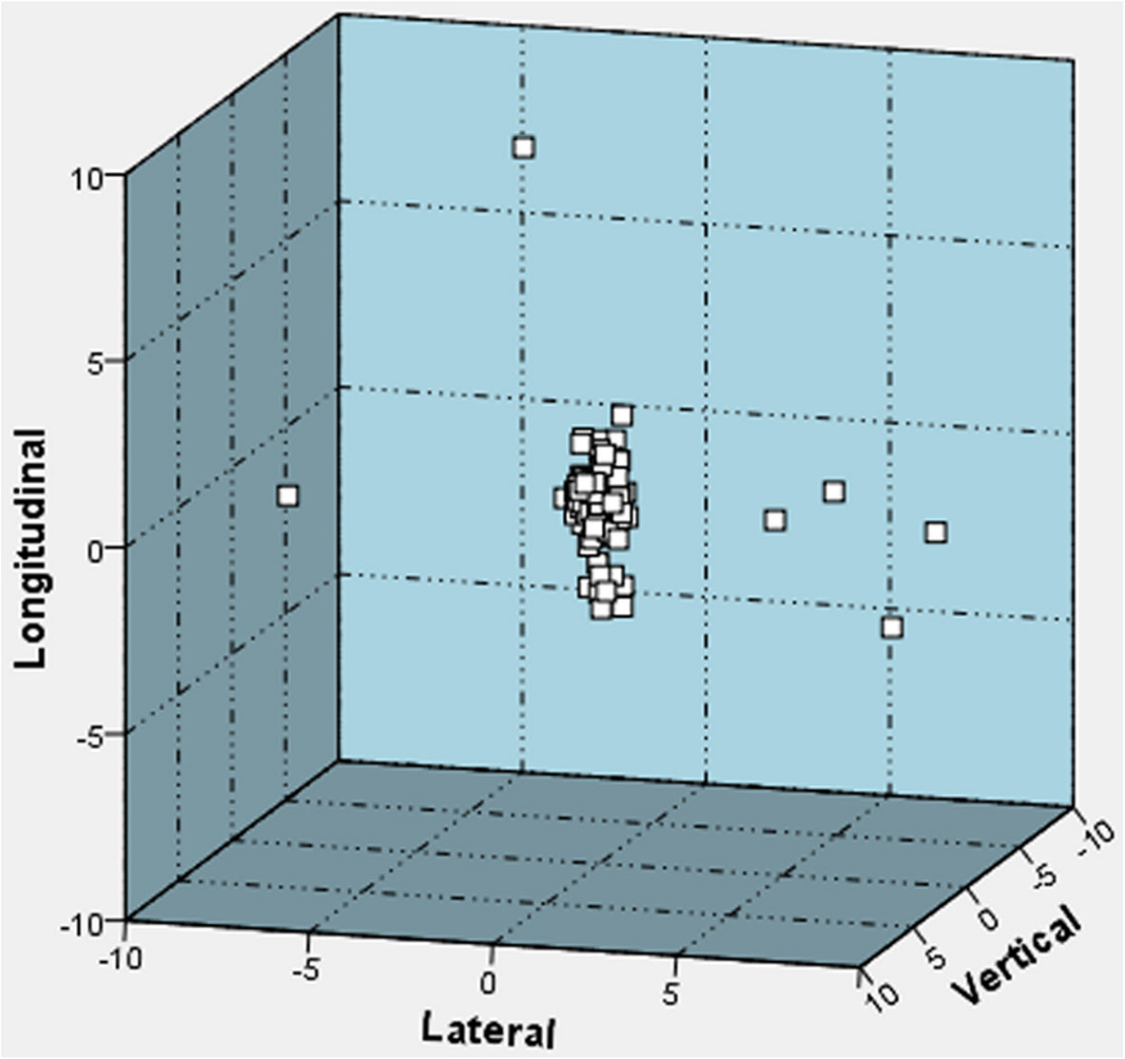
Online adjustments after cone beam computed tomography scans with low pressure foil [cm]

**Fig. 3:**
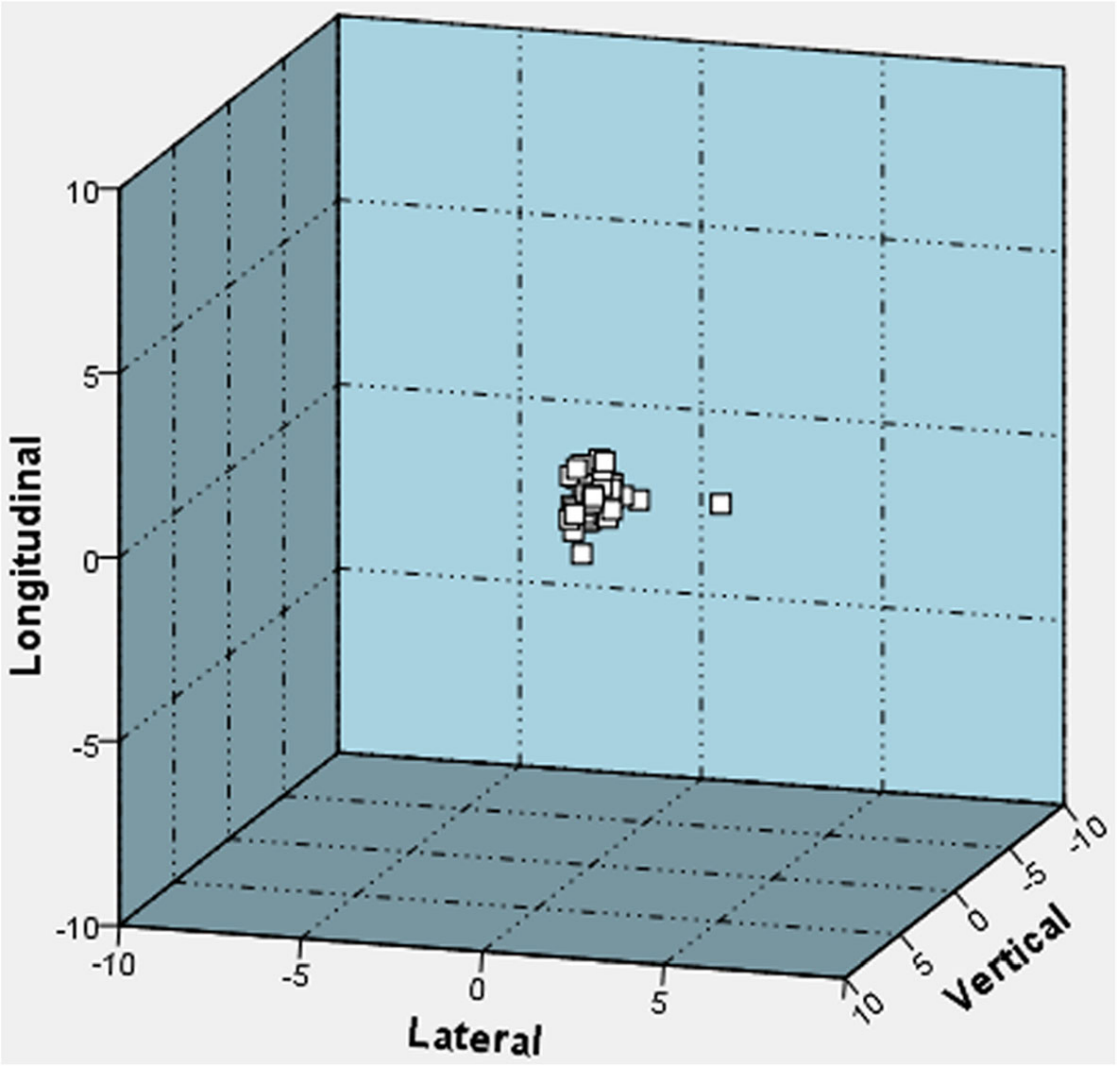
Online adjustments after cone beam computed tomography scans with abdominal compression [cm]

The online adjustments were evaluated in general and with regard to its absolute values, thus leading to a comparison of the true amount of movements. The mean online adjustments in the vertical, lateral and longitudinal axis account for − 0.11 cm (±0.40 cm), 0.16 cm (±0.95 cm) and − 0.28 cm (±0.87 cm) with low pressure foil and 0.09 cm (±0.43 cm), 0.16 cm (±0.29 cm) and 0.08 cm (±0.44 cm) with abdominal compression (Figs. 2 and 3).

The absolute mean value of the online adjustments in the vertical, lateral and longitudinal axis account for 0.29 cm (±0.50 cm), 0.48 cm (±1.50 cm) and 0.57 cm (±0.89 cm) with low pressure foil and 0.28 cm (±0.28 cm), 0.29 cm (±0.43 cm) and 0.33 cm (±0.32 cm) with abdominal compression, respectively. The vertical, lateral and longitudinal online adjustments were statistically significant different between both groups (*p* < 0.013). The absolute values of the online adjustments were statistically different in case of the longitudinal axis (*p* = 0.043).

Summarizing the adjustments in vectors results in a mean vector of 0.99 cm (±1.59 cm) with low pressure foil and 0.72 cm (±0.46 cm) with abdominal compression. There was no statistically significant difference of the vectors neither between the two groups, nor between the vectors of two fractions within one group.

## Discussion

Robust treatment planning is based on contouring an ITV, which takes into account the maximum extent of tumor motion. Liver motion is due to the breathing amplitude, which should be minimized by an appropriate patient immobilization. There are a lot of possible ways of patient immobilization in SBRT, but low pressure foil and abdominal compression are highly promising, easy to use, with little interfractional displacements and characterized by good reproducibility [12–15]. In our results, there was not any significant difference in case of the GTV/ITV ratio in both groups. This may be explained on the one hand by the small collective of this study, the heterogeneous body mass index and on the other hand by the tumor movement itself. On top of that, this is a retrospective study and the group with abdominal compression was smaller than the group with low pressure foil. Another explanation might be the big difference of the gross tumor volumes between both groups. With the GTV being bigger in the group with low pressure foil, the ratio of GTV/ITV might possibly be even smaller in reality (with similar volumes in both groups), thus leading to a significant difference between both groups. This assumption is most likely true as the real tumor movement in the group of abdominal compression compared to the group of low pressure foil was smaller in every direction. We could even show a significant difference in case of cranial movement.

However, the extent of online corrections showed a statistically significant difference between both groups. Abdominal compression leads to a shift in overall liver motion which is especially due to a decreased motion in the longitudinal axis and a compensating motion away from the abdominal compression axis. The fact that a change in patient immobilization mainly leads to a displacement shift with one dominant direction is represented in our evaluation of the 4D-CTs and could also be shown in a similar study about SBRT of lung tumors [16]. Nevertheless, the significant change in one main direction is probably compensated by the other directions and leads to a compensation in case of an ITV-generation. This can also explain the significant differences in each direction of online adjustments, whereas the mean values of online adjustments are only statistically significantly different in the longitudinal axis but not in the other dimensions. Online adjustments, that is to say the precision of patient positioning can be improved by the usage of abdominal compression but not in every setting. The values with but not without abdominal compression match with the results from a study from Herfarth et al., showing that this is a feasible way of patient immobilization, providing high accuracy [17]. Together with the studies from Herfarth et al. and Navarro-Martin et al. the impact of immobilization on interfractional robustness and setup reproducibility in general could be shown [16, 17]. Nevertheless, these studies about immobilization in SBRT are based on rigid immobilization setups. Promising results about frameless SBRT could be shown for lung cancer [18–20]. In case of liver cancer, rigid immobilization setups, such as abdominal compression, are still the most established safety devices to ensure robust and accurate SBRT. This gain in accuracy is mainly dependent on adjustments according to CBCT. But, online matching by the use of CBCT has to be critically evaluated, as matching is based on the analysis of the liver’s soft tissue. CBCT does not allow safe identification of the target volume. Online adjustments are therefore based on the total liver volume but not the target volume. But Eccles et al. could show that the interfractional change or variability of the liver volume is small and therefore reliable [21]. However, fiducial markers such as lipiodol or gold fiducials are highly promising and effective in case of image-guided radiotherapy [22, 23]. Nevertheless, these markers can only be used by invasive interventions prior to radiotherapy, coming along with distinct complications, which is why other principles of guiding are the topic of present studies [24].

With regard to robust treatment planning, breathing is summarized in the treatment planning CT scan by both inspiration and expiration – no breath-hold was performed during CT scanning. The robustness of this CT scan is ensured by an ITV increasing the accuracy of treatment application. With the CBCT scan again being performed with both breathing cycles, the registration of the CBCT on the treatment planning CT scan can be done. The robustness of this kind of registration could be increased by gating, that is to say irradiating only during expiration or inspiration, thus reducing the impact of tumor motion [25, 26].

Tracking, that is to say following the tumor motion is another method of robust irradiation with highly precise dose application in the tumor volume. Unfortunately, there is still little knowledge on the best conditions of tracking, which is why future studies have to show its feasibility [27].

## Conclusion

In summary, clinical practice in radiotherapy of liver tumors is still mainly based on SBRT with distinct methods of patient positioning and reducing tumor motion. Generation of an ITV and patient immobilization in our study could show its feasibility and the potential advantage of abdominal compression in contrast to immobilization by low pressure foil. By increasing robustness the general underuse of SBRT in the therapy of primary and secondary liver cancer can possibly be overcome and SBRT will not only be used in case of a big tumor volume or mainly venous blood supply with only little arterial vessels supplying the tumor [28, 29]. In these cases, SBRT is beneficial and promising, both in palliative therapy and in case of bridging to liver transplantation. But by increasing robustness and accuracy of radiotherapy, the probability of limiting complications, such as RILD and a general decrease in liver function, will further be reduced and the indications of SBRT will close up to those of selective internal radiation therapy, radiofrequency ablation and transarterial chemoembolization [30].

## Abbreviations

CBCT: Cone beam computed tomography
CT: Computed tomography
GTV: Gross tumor volume
ITV: Internal target volume
RILD: Radiatiotherapy-induced liver disease
RPM: Respiratory position management
SBRT: Stereotactic body radiotherapy
SPSS: Statistical package for the social sciences
TPS: Treatment planning system
VMAT: Volumetric modulated arc therapy
